# Acceptance and Uptake of Technology Among Older Adults: Findings From CREATE

**DOI:** 10.1093/geroni/igaa057.1820

**Published:** 2020-12-16

**Authors:** Sara Czaja, Bo Xie

## Abstract

Technology is ubiquitous in everyday life and technology use is essential to independent living. Technology applications are deployed in most settings (e.g., healthcare, work) and also changing how we communicate, and access information and services. Despite increases in technology uptake among older people, an age-related digital divide remains. Drawing from research conducted by the Center for Research and Education on Aging and Technology Enhancement (CREATE), this symposium will discuss the acceptance and uptake of technology among older adults and factors influencing technology adoption. S. Czaja will discuss recent technology trends and how they vary according to technology type and subgroups of older adults. Based on CREATE findings, she will also discuss age group and cohort differences in interest in and comfort with technology. W. Boot will present findings from a study that examined adherence to a technology-based cognitive training program and how individual difference factors shaped adherence. N. Charness will present findings regarding whether advanced driver assistance systems (ADAS) can improve older adult driving performance, and older adults’ acceptance and perceptions of value of ADAS systems. M. Harris will discuss health related technology interventions and how integration of technology acceptance and behavior change models can provide insights for the design of health behavior interventions aimed at older adults. J. Sharit will provide findings from a study, which examined the willingness of older adults to adopt a variety of technologies and factors influencing willingness to adopt. Bo Xie will lead a discussion of these issues and outline areas for future research.

